# Systematic evaluation and meta-analysis of the efficacy of Jingjin acupuncture therapy in the treatment of peripheral facial palsy

**DOI:** 10.3389/fneur.2024.1459738

**Published:** 2024-12-18

**Authors:** Xingyu Kang, Ying Huang, Xueyan Lv, Xiaofang Liu, Siyu Chen, Le Ma, Shuai Shi

**Affiliations:** ^1^Graduate School, Heilongjiang University of Chinese Medicine, Harbin, China; ^2^Oncology Department, The Second Affiliated Hospital of Heilongjiang University of Chinese Medicine, Harbin, China; ^3^Rehabilitation Department, The Second Affiliated Hospital of Heilongjiang University of Chinese Medicine, Harbin, China

**Keywords:** Jingjin, meridian, sinew, acupuncture, facial paralysis

## Abstract

**Objective:**

This study aimed to systematically evaluate the clinical efficacy of Jingjin (muscle region of the meridian, sinew/tendon/fascia) acupuncture therapy in treating peripheral facial paralysis.

**Methods:**

A computerized search of PubMed, EMBASE, Cochrane Central Register of Controlled Clinical Studies, SCOPUS, Web of Science, PEDro, China Knowledge, Wanfang, and Wipu databases was performed for published randomized controlled trials (RCTs) on the treatment of peripheral facial paralysis using Jingjin acupuncture therapy from the beginning of the construction of the databases until 2 April 2024. After a two-person independent extraction of data, the studies were assessed for paper quality and then analyzed for meta-analysis using RevMan5.4 software.

**Results:**

A total of 19 randomized controlled trials involving 1,436 patients were included. Meta-analysis showed that Jingjin acupuncture therapy for peripheral facial palsy had a higher overall effectiveness rate (OR = 3.93, 95% CI [2.78, 5.56], Z = 7.75, *p* < 0.00001), and cure rates (RR = 1.69, 95% CI [1.51, 1.90], and Z = 8.89, *p* < 0.00001) were higher than those of conventional therapy. Jingjin acupuncture therapy was also superior to conventional acupuncture therapy in terms of Facial Disability Index-Physical (FDIP) scores, Facial Disability Index-Social (FDIS) scores, facial nerve function scores, and Portmann scores on the Facial Disability Index Scale in patients with peripheral facial paralysis.

**Conclusion:**

Jingjin acupuncture therapy is effective in treating peripheral facial paralysis and has better overall efficacy than conventional therapy. However, the reliability is limited by the small number of high-quality studies with scientifically rigorous methods and designs, so more large-sample, high-quality, randomized controlled studies are still needed for further validation.

**Systematic review registration:**

https://www.crd.york.ac.uk/prospero/, Identifier CRD42024543195.

## 1 Introduction

Peripheral facial paralysis (PFP) is a type of peripheral paralysis of the facial nerve, which can be triggered by viral infections, local infections, trauma, immune system disorders, tumors, and other factors (1, 2). The symptoms are mainly manifested as dysfunction of the muscles of expression on one side of the face (3), including loss of facial expression, loss of facial lines, inability to frown and close the eyes, shallow nasolabial folds, mouth drooping, cheek funneling, drooping, patient of the cheeks, and air leakage (4). These symptoms significantly impact the quality of life of patients (5). Western medicine mainly uses hormones, nutritive nerve drugs, and botulinum toxin injections, but they produce drug resistance and adverse reactions (6, 7).

Jingjin acupuncture therapy (8) (muscle region of the meridian, sinew/tendon/fascia), a form of traditional Chinese medicine, provides a new way of thinking about the treatment of peripheral facial paralysis. Unlike traditional acupuncture, which is based on the meridian theory that posits the existence of 12 meridians and 8 extra meridians in the human body, Jingjin acupuncture therapy is based on the Jingjin theory. Traditional acupuncture seeks to treat diseases by needling the acupoints on these meridians (9). In contrast, Jingjin acupuncture focuses on the 12 meridians of the human body, which are connected to the external system of tendons and joints. Jingjin acupuncture therapy emphasizes the principle of “pain as acupoint,” meaning that acupuncture points are selected according to the pain points. The therapy focuses on directly treating the lesions of the muscle system. Jingjin acupuncture therapy, through the precise positioning of the facial meridian and deep stimulation, can be more effective in improving facial nerve function (10). Under the guidance of the theory of “Jingjin,” later generations have continuously invented and developed needles and needling methods, so the method of Jingjin acupuncture therapy has made significant progress, which includes various kinds of needles (needle knives, sharp-edged needles, circular needles, and other needling methods) and needling methods (Qi needling, Hegu needling, filiform needling, etc.). (11). In recent years, the number of related clinical studies (12) has been increasing yearly, but systematic evaluations and meta-analyses of associated studies are rarely seen. In this study, we searched the relevant databases for clinical randomized controlled trials on the treatment of peripheral facial palsy using Jingjin acupuncture therapy, performed a quality assessment and meta-analysis of the included literature, evaluated the efficacy of Jingjin acupuncture therapy in treating peripheral facial palsy at different stages, and consolidated and analyzed the outcome indices, to provide evidence-based medical support for the treatment of peripheral facial palsy using Jingjin acupuncture therapy.

## 2 Materials and methods

### 2.1 Study design

This study protocol has been registered in PROSPERO, and the registration number is CRD42024543195. Our study was conducted per the Preferred Reporting Items for Systematic Reviews and Meta-Analyses (PRISMA) Reporting Guidelines. This meta-analysis followed the guidelines provided in the Preferred Reporting Items for Systematic Reviews and Meta-Analyses, and the study design was based on the PICO principles. P-Participants/population: Participants had to meet the clinical diagnosis of peripheral facial palsy, and there were no restrictions on age, gender, or ethnicity. I-Intervention: The study group received Jingjin acupuncture therapy in addition to the control group; C-Comparison group/control group: The control group did not receive Jingjin acupuncture therapy and the rehabilitation treatment modality was not limited; O-Outcome: the primary outcome indices included the overall efficacy rate, cure rate, Facial Disability Index-Physical (FDIP) (13), Facial Disability Index-Social (FDIS) (14), facial nerve function scores (15, 16), and Portmann scores (17).

### 2.2 Search strategy

A literature search in both Chinese and English was conducted using a combination of subject terms and free words. The Chinese search terms included: “针,” “刺,” “经筋,” “面瘫,” “面神经麻痹,” “特发性面神经麻痹,” “面神经炎,” “贝尔氏麻痹,” and “贝尔面瘫.” The English search terms included: “Needle,” “Prick,” “Jingjin,” “Meridian Sinew,” “Facial paralysis,” “Facial nerve palsy, ““Idiopathic facial nerve palsy,” “Facial neuritis,” “Bell’s palsy,” and “Bell’s facial palsy.” Computerized searches of the PubMed database, EMBASE, Cochrane Central Register of Controlled Clinical Studies, SCOPUS, Web of Science, PEDro, China Knowledge, Wanfang, and Wipro for published randomized controlled trial (RCT) studies on the treatment of cerebral palsy with Jingjin acupuncture therapy from the beginning of the databases until 2 April 2024, were gathered. The collected literature was then cross-searched and screened for literature that met the inclusion criteria.

### 2.3 Inclusion criteria

(1) Study type: The study had to be a randomized controlled trial (RCT), with the language of the literature either Chinese or English, and no restriction on the time of publication, regardless of whether blinding or allocation concealment was used. (2) Study population: The original literature had to have clear Western or Chinese medicine diagnostic criteria to confirm the diagnosis of peripheral facial paralysis patients (2); ethnicity, age, gender, and other baseline characteristics were not restricted but had to be comparable. (3) Interventions: The treatment group received Jingjin acupuncture therapy, while the control group was treated with conventional therapies, including conventional acupuncture therapy, rehabilitation therapy, and drug therapy. The treatment group also received a combination of conventional rehabilitation therapy and acupuncture therapy. (4) Outcome evaluation indices: The outcome measures included total efficacy rate, cure rate, Facial Disability Index-Physical (FDIP), Facial Disability Index-Social (FDIS), facial nerve function scores, and Portmann scores.

### 2.4 Exclusion criteria

Type of study: non-RCT studies, case reports, empirical summaries, own before and after controls, and review literature were excluded. (2) Study population: non-peripheral facial paralysis cases and animal experiment-type literature were excluded. (3) Interventions: Literature in which the therapeutic measures involved other treatments that affected the causal interpretation of the final treatment were excluded. (4) Efficacy evaluation: Literature with non-standardized efficacy evaluation indices were excluded. (5) Repeated publication of data from the same trials, literature of poor quality (e.g., excessive risk of bias and small sample size), and literature with unclear reporting of the original literature were excluded. (6) Literature with unclear diagnoses of peripheral facial paralysis or comorbidities with other diseases was excluded. (7) Literature with unclear findings, incomplete data, or unsuccessful contact with full-text authors was excluded.

### 2.5 Data extraction and risk of bias assessment

Studies were independently searched and screened by two evaluators, and then the results were compared by a third reviewer in case of disagreement. The content included first author, year of publication, country, age (treatment and control groups), duration of disease (treatment and control groups), number of cases (treatment and control groups), interventions (treatment and control groups), test parameters, duration of treatment, and consistency at baseline. The quality of the literature was assessed using the “risk of bias assessment” tool recommended by the Cochrane Handbook of Systematic Reviews, which evaluates the risk of bias for seven items, including generation of randomized sequences, concealment of allocation, blinding of investigators, and subjects, blinding of study endpoints, completeness of endpoints, selective reporting of results, and other biases. Bias. Each entry was assessed for risk of bias and categorized as high, low, or uncertain.

### 2.6 Grade evaluation

The GRADE methodology assessed the quality of evidence and the strength of the recommendations (18). The GRADE methodology considers the following five elements: risk of bias, inconsistency, indirectness, inaccuracy, and publication bias. The outcomes of the included literature were categorized as very low, low, moderate, and high quality based on the elements assessed.

### 2.7 Data analysis

For meta-analysis of the data included in the study, RevMan 5.4 software was utilized. For dichotomous data effect sizes were combined using odds ratios (ORs) and their 95% confidence intervals (CIs). Continuous variable data were combined statistically using mean difference (MD) or standardized mean difference (SMD). The effect model could be either a random-effects or a fixed-effects model, with the effect sizes expressed through 95% CIs. The fixed-effects model in meta-analysis needs to meet the condition of no statistical heterogeneity between the data of each study (*p* ≥ 0.1 and I^2^ ≤ 50%). Heterogeneity between the data of each study was analyzed using I^2^ and *p*-values. If I^2^ ≤ 50%, the fixed-effects model was used; otherwise, sensitivity analyses were performed, and then either the fixed-effects model or the random-effects model was used directly. Publication bias was assessed using a funnel plot, with symmetrical graphs indicating no publication bias and vice versa.

## 3 Results

### 3.1 Results of the literature search

**Figure 1:**
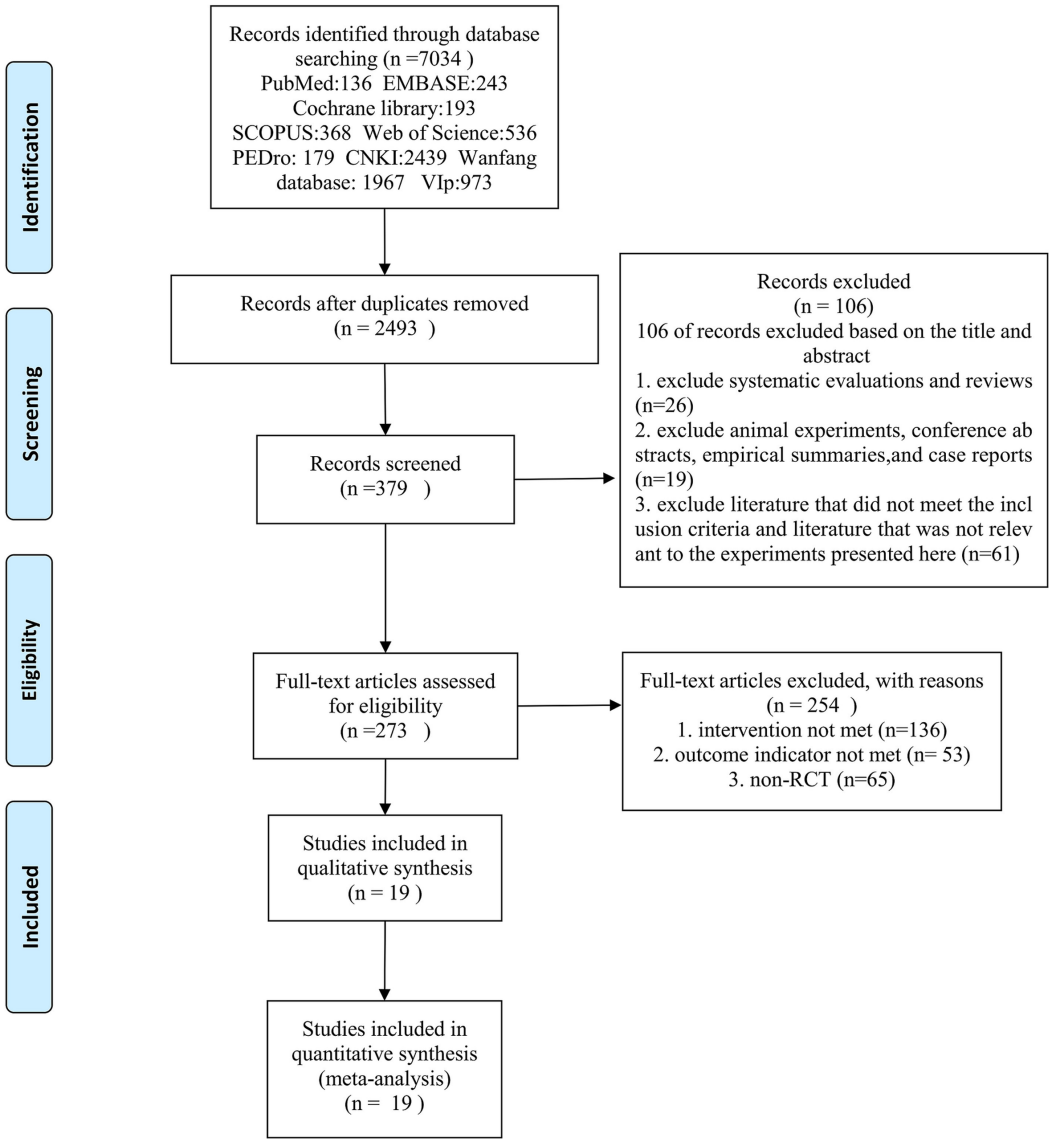
Flowchart of literature screening.

A total of 7,034 studies were searched in 9 databases; after checking and reading the titles and abstracts, 379 studies were initially screened. After full-text screening, the documents that did not meet the screening conditions were excluded. Finally, 19 studies were included, and the literature screening process is shown in Figure 1.

### 3.2 Basic characteristics of the included studies

All 19 trials were single-center clinical RCTs, with 1,436 cases ranging from 32 to 200 patients in each study. The included literature met the predefined inclusion and exclusion criteria, and Table 1 shows the main characteristics of all the original studies.

**Table 1:** Basic characteristics of the included studies.

First author	Year	Country	Age/years		Disease course		Sample size		Intervention		Outcome	Intervention time	Baseline consistency
			T	C	T	C	T	C	T	C		d	
CLC	2022	China	32.29 ± 12.04	32.54 ± 13.27	(6.35 ± 1.53)d	(6.24 ± 1.12)d	30	30	JA	RR	①②	20	Concordance
HMT	2022	China	44.69 ± 13.535	45.19 ± 13.642	(7.19 ± 2.822)m	(6.94 ± 2.355)m	32	32	JA	RR	①②④⑤	30	Incoherence
LDM	2021	China	38.37 ± 12.51	38.10 ± 12.50	(17.17 ± 7.39)d	(15.17 ± 6.88)d	30	30	JA	RR	①②④⑤	28	Concordance
LSY	2008	China	14 ~ 70	14 ~ 70	(1 ~ 30)d	(1 ~ 30)d	50	50	JA	RR	①②	10	Concordance
LCC	2015	China	48.5 ± 3.73	46.2 ± 5.14	(7.65 ± 0.8)d	(8.49 ± 1.2)d	30	30	JA	RR	①②	30	Concordance
LF	2022	China	49.72 ± 14.724	48.47 ± 16.274	(18.53 ± 2.016)d	(18.38 ± 2.121)d	33	33	JA	RR	①②③④⑤	28	Concordance
LHM	2002	China	3 ~ 68	5 ~ 70	(1 ~ 180)d	(1 ~ 150)d	54	51	JA	RR	①②	10	Concordance
LL	2022	China	50.52 ± 13.09	46.27 ± 12.12	(73.48 ± 13.76)d	(75.73 ± 11.43)d	21	22	JA	RR	①②⑤	30	Concordance
MJ	2020	China	42.13 ± 13.559	39.90 ± 14.587	(3.27 ± 1.507)d	(3.73 ± 1.760)d	30	30	JA	RR	①②③④	28	Concordance
RJG	2018	China	39.828 ± 12.509	39.806 ± 12.359	(10.150 ± 2.512)d	(10.060 ± 2.605)d	34	34	JA	RR	①②	30	Concordance
TXY	2015	China	55.15 ± 13.29	53.41 ± 17.70	(30 ~ 90)d	(30 ~ 90)d	16	16	JA	RR	①②	28	Concordance
WDB	2009	China	31.75 ± 5.45	33.75 ± 5.05	(9 ± 6.7)d	(10 ± 5.9)d	43	43	JA	RR	①②	20	Concordance
WFD	2013	China	16 ~ 76	16 ~ 76	(1 ~ 60)d	(1 ~ 60)d	100	100	JA	RR	①②	10	Concordance
XZH	2022	China	40.32 ± 13.79	41.40 ± 11.75	(1 ~ 7)d	(1 ~ 7)d	30	30	JA	RR	①②⑤⑥	20	Concordance
YXQ	2021	China	43.71 ± 14.664	45.15 ± 13.517	(8.87 ± 0.935)d	(8.56 ± 0.718)d	40	40	JA	RR	①②⑤⑥	5	Concordance
YGW	2022	China	32.53 ± 6.75	33.16 ± 7.08	(3.32 ± 1.65)d	(3.27 ± 1.48)d	41	41	JA	RR	①②	20	Concordance
YXX	2023	China	40.69 ± 5.31	40.71 ± 5.06	(5.13 ± 0.69)d	(5.04 ± 0.63)d	30	30	JA	RR	①②⑤⑥	18	Concordance
YX	2009	China	40.12 ± 10.64	43.36 ± 10.21	4 h ~ 6d	12 h ~ 7d	50	50	JA	RR	①②③④	20	Concordance
ZZT	2016	China	24 ~ 58	23 ~ 60	(2 ~ 180)d	(5 ~ 180)d	27	23	JA	RR	①②	42	Incoherence

T: test group; C: control group; d: day; m: month. ① Total efficacy rate; ② Ture rate; ③ FDIP; ④ FDIS; ⑤ Facial Nerve Function Score; and ⑥ Portmann. JA = Jingjin acupuncture, RR = Routine rehabilitation.

### 3.3 Study risk of bias assessment

**Figure 2:**
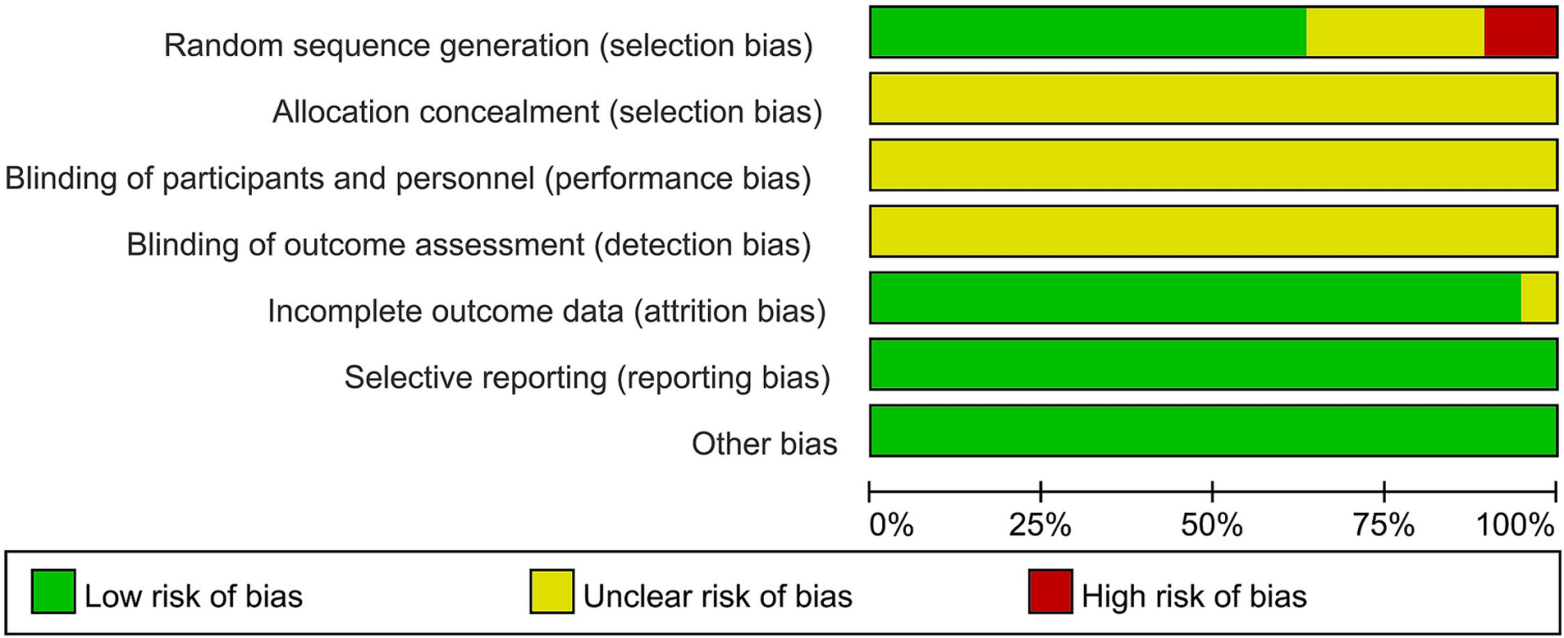
Risk of bias assessment chart.

**Figure 3:**
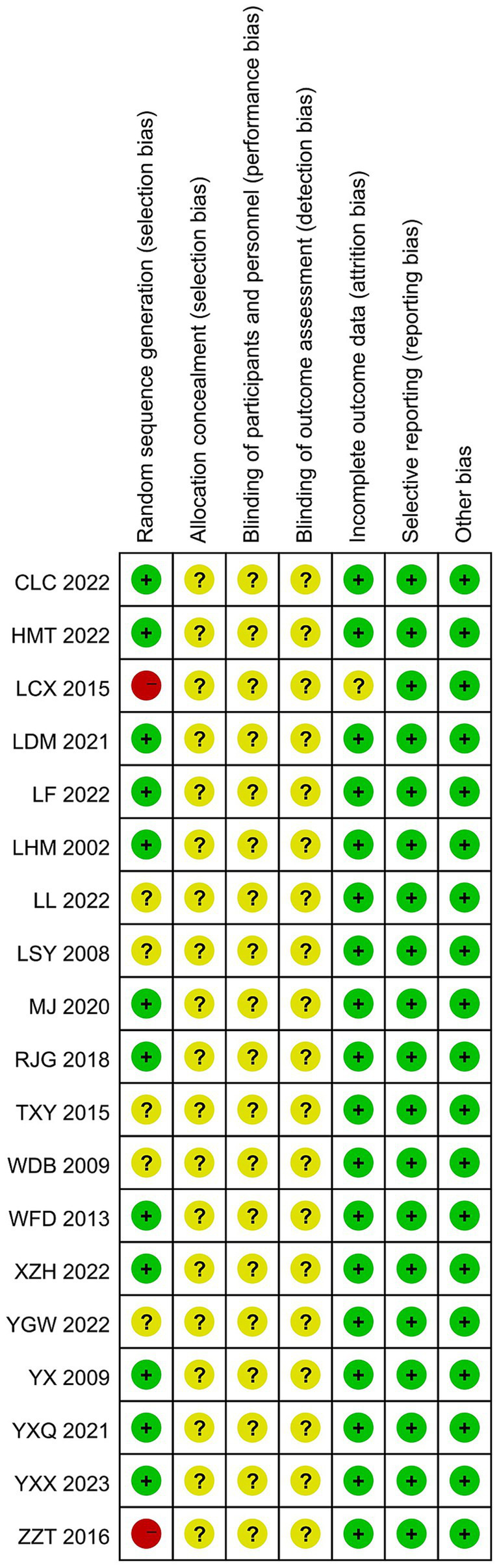
Risk of bias assessment chart.

A total of 14 of the 19 studies specifically described the method of generating the randomized sequence, 11 (19–36) used randomized numerical tables, 2 (23, 37) used the order-of-attendance grouping method, which was judged to be “high risk,” and 1 (31) used the lottery method, while the remaining studies described only randomized grouping in an unspecified manner and rated them as “unknown risk.” With regard to concealment of protocol allocation, four (24, 27, 32, 33) studies mentioned concealment of protocol allocation, and blinding was not explicitly applied in any of them. A total of 19 studies had complete data, and selective reporting was complete and unbiased. Regarding other biases, two (20, 37) studies did not report whether baseline information was comparable. Charts to assess the risk of bias in the literature were generated by RevMan 5.4 software, as shown in Figures 2, 3.

### 3.4 Analysis of outcome indicators

#### 3.4.1 Overall efficiency

**Figure 4:**
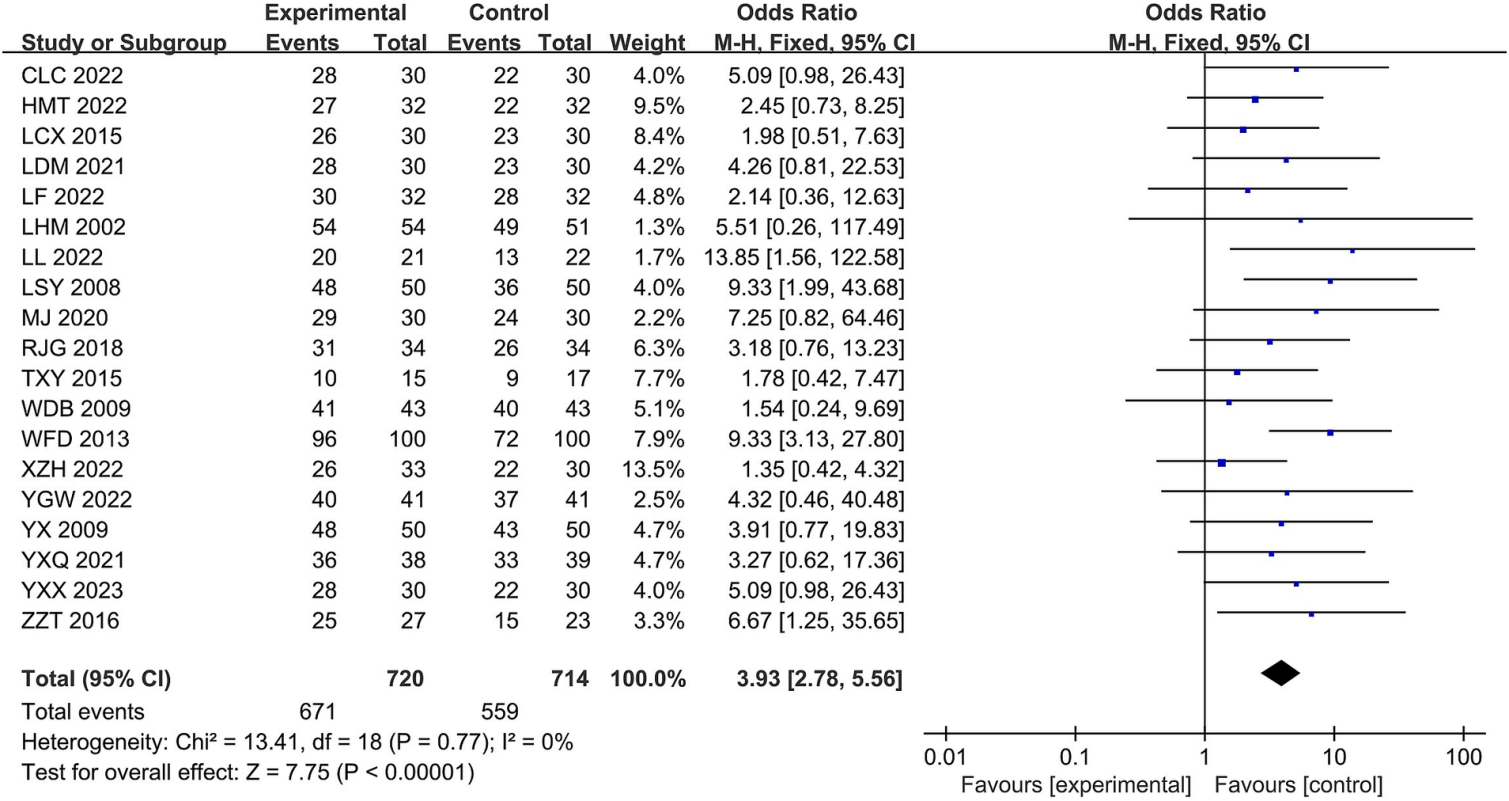
Forest plot of the total efficiency rate.

A total of 19 studies reported overall effectiveness rates. The results of the heterogeneity analysis, χ^2^ = 13.41, *p* = 0.77, I^2^ = 0%, indicated that the statistics of these randomized trials were homogeneous. Therefore, a fixed-effects model was used statistically, with an overall OR = 3.93, 95% CI of [2.78, 5.56], and a combined-effects test, Z = 7.75, *p* < 0.00001, indicating a statistically significant difference. In terms of total efficacy rate, the Jingjin acupuncture therapy was superior to conventional therapy (see Figure 4).

#### 3.4.2 Cure rate

**Figure 5:**
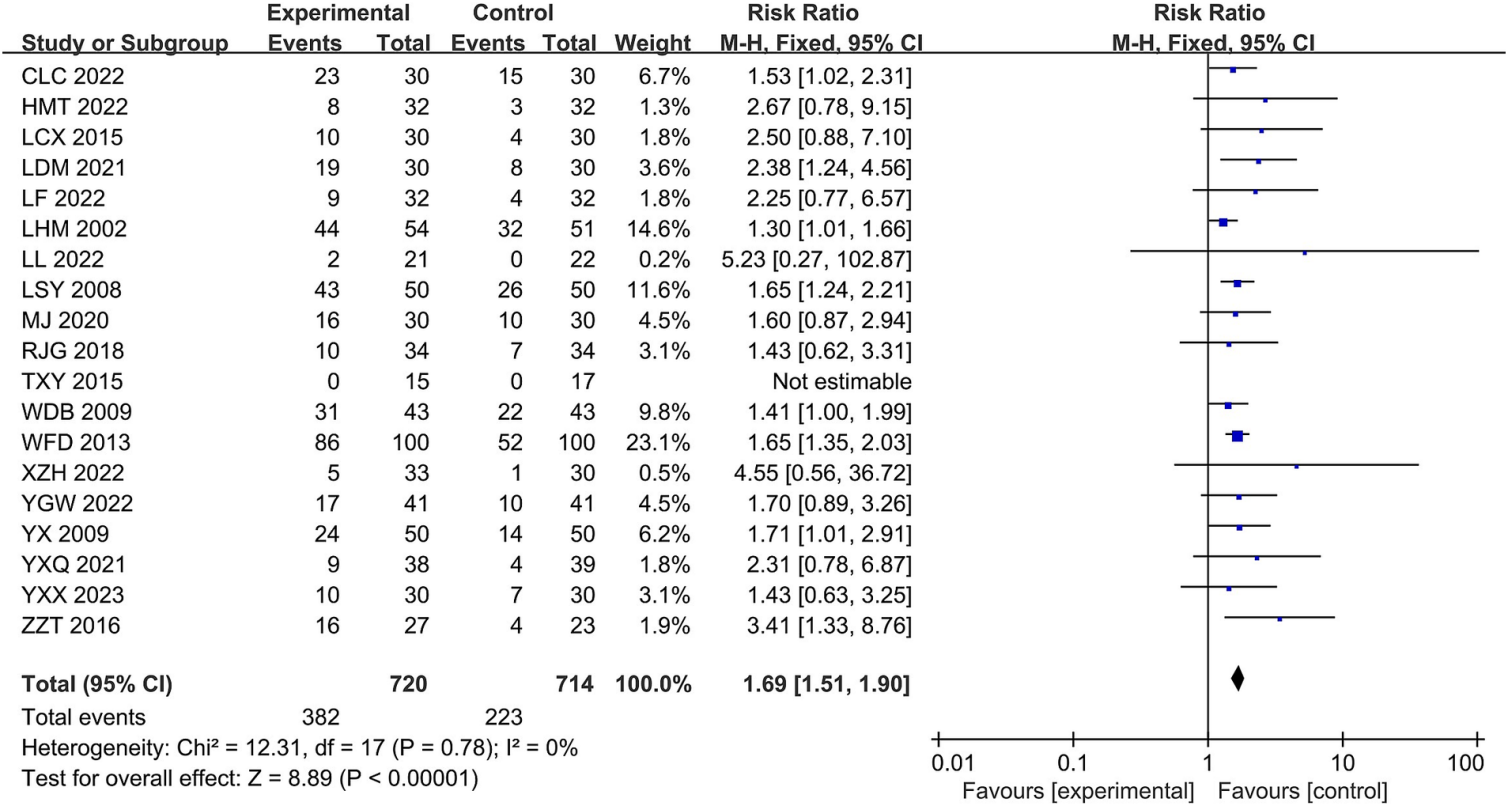
Forest plot of cure rates.

All 19 studies reported cure rates. The heterogeneity among the RCTs in the 19 studies was χ^2^ = 12.31, *p* = 0.78, and I^2^ = 0%, which was homogeneous and therefore analyzed using the fixed-effects model. The total RR was 1.69, with a 95% CI of [1.51, 1.90]. The combined effect size test yielded Z = 8.89, *p* < 0.00001, indicating a statistically significant difference. In terms of cure rate, the Jingjin acupuncture therapy was superior to conventional treatment (see Figure 5).

#### 3.4.3 Facial disability index-physical

**Figure 6:**
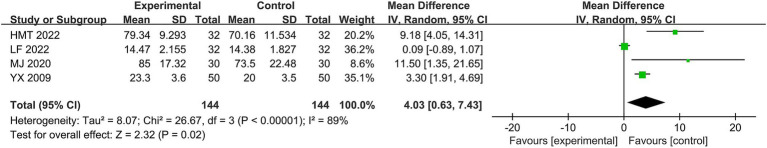
Forest plot of the FDIP scale.

The four studies (20, 24, 27, 36) were analyzed using FDIP scores. The heterogeneity of the four literature RCTs was χ^2^ = 26.67, *p* < 0.00001, and I^2^ = 89%, which is highly heterogeneous, so a random-effects model was used to analyze the results. The total MD was 4.03, with a 95% CI of [0.63, 7.43], and the combined effect size test revealed a statistically significant difference (Z = 2.32, *p* = 0.02) (see Figure 6). To analyze the source of heterogeneity, the sensitivity analysis was conducted by excluding the literature one by one method and excluding any literature. Heterogeneity remained high, so subgroup analyses were attempted. The subgroup analyses were conducted based on disease duration, sample size, and specific differences in interventions, among others, to assess the impact of these factors on outcomes. However, the subgroup analyses failed to significantly reduce heterogeneity, suggesting that other unidentified factors may have influenced the results. The scores of the treatment group were higher than those of the control group, which indicated that the Jingjin acupuncture therapy was superior to the conventional therapies in terms of the FDIP scores.

#### 3.4.4 Facial disability index-social

**Figure 7:**
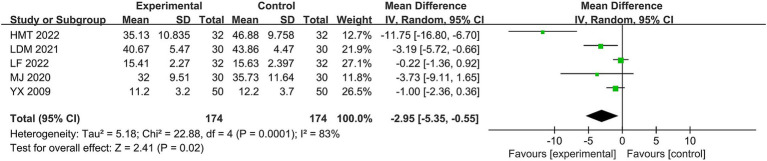
Forest plot of the FDIS scale.

Five studies (20, 21, 24, 27, 36) used the FDIS scores. The heterogeneity of the RCTs of the five studies was χ^2^ = 22.88, *p* = 0.0001, I^2^ = 83%, which was highly heterogeneous and therefore analyzed using the random-effects model. The difference was statistically significant for the total MD = −2.95, 95% CI [−5.35, −0.55], combined effect size test, Z = 2.41, *p* = 0.02 (see Figure 7). To analyze the source of heterogeneity, we used the same method for consistency as we did for the FDIP scores; unfortunately, sensitivity and subgroup analyses failed to significantly reduce the heterogeneity, suggesting that other unidentified factors may have influenced the results. The scores of the treatment group were higher than those of the control group, and the overall results indicated that the Jingjin acupuncture therapy was superior to the conventional therapies in evaluating FDIS scores in the treatment of peripheral facial paralysis.

#### 3.4.5 Facial nerve function

**Figure 8:**
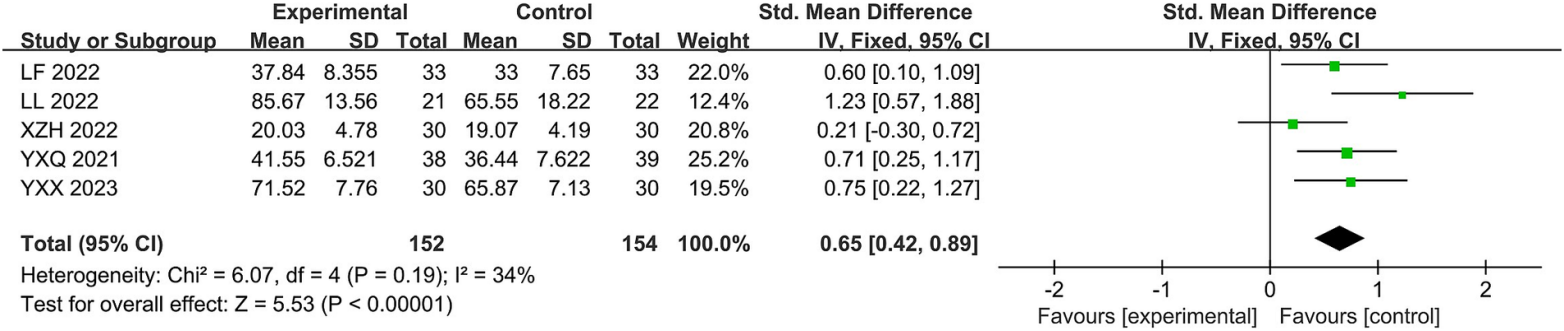
Forest plot of the facial nerve function scale.

Five studies (24, 32, 33, 35, 36) used facial nerve function scores. The heterogeneity of the RCTs of the five studies was χ^2^ = 6.07, *p* = 0.19, and I^2^ = 34%, with mild heterogeneity, so it was analyzed using a fixed-effects model. Despite the different scales used to evaluate facial nerve function, the total SMD was 0.65, with a 95% CI of [0.42, 0.89]. The combined effect size test yielded Z = 5.53, *p* < 0.00001, indicating a statistically significant difference. The facial nerve function evaluation of Jingjin acupuncture therapy for peripheral facial paralysis was superior to the conventional treatment (see Figure 8).

#### 3.4.6 Portmann

**Figure 9:**
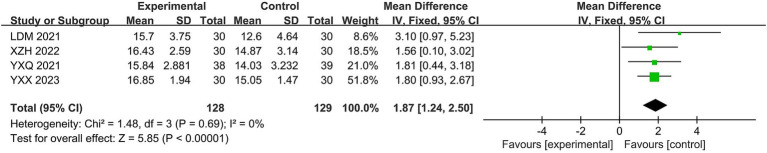
Forest plot of Portmann scores.

Four studies (21, 32, 33, 35) were analyzed using the Portmann scores. The heterogeneity of the RCTs was χ^2^ = 1.48, *p* = 0.69, and I^2^ = 0%, which was homogeneous and therefore analyzed using a fixed-effects model. The total MD was 0.65, with a 95% CI of [0.42, 0.89]. The combined effect size test yielded Z = 5.53, *p* < 0.00001, indicating a statistically significant difference. In Portmann’s evaluation of Jingjin acupuncture therapy for peripheral facial paralysis, the Jingjin acupuncture therapy was superior to the conventional treatment (see Figure 9).

### 3.5 Inclusion of literature publication Bias

**Figure 10:**
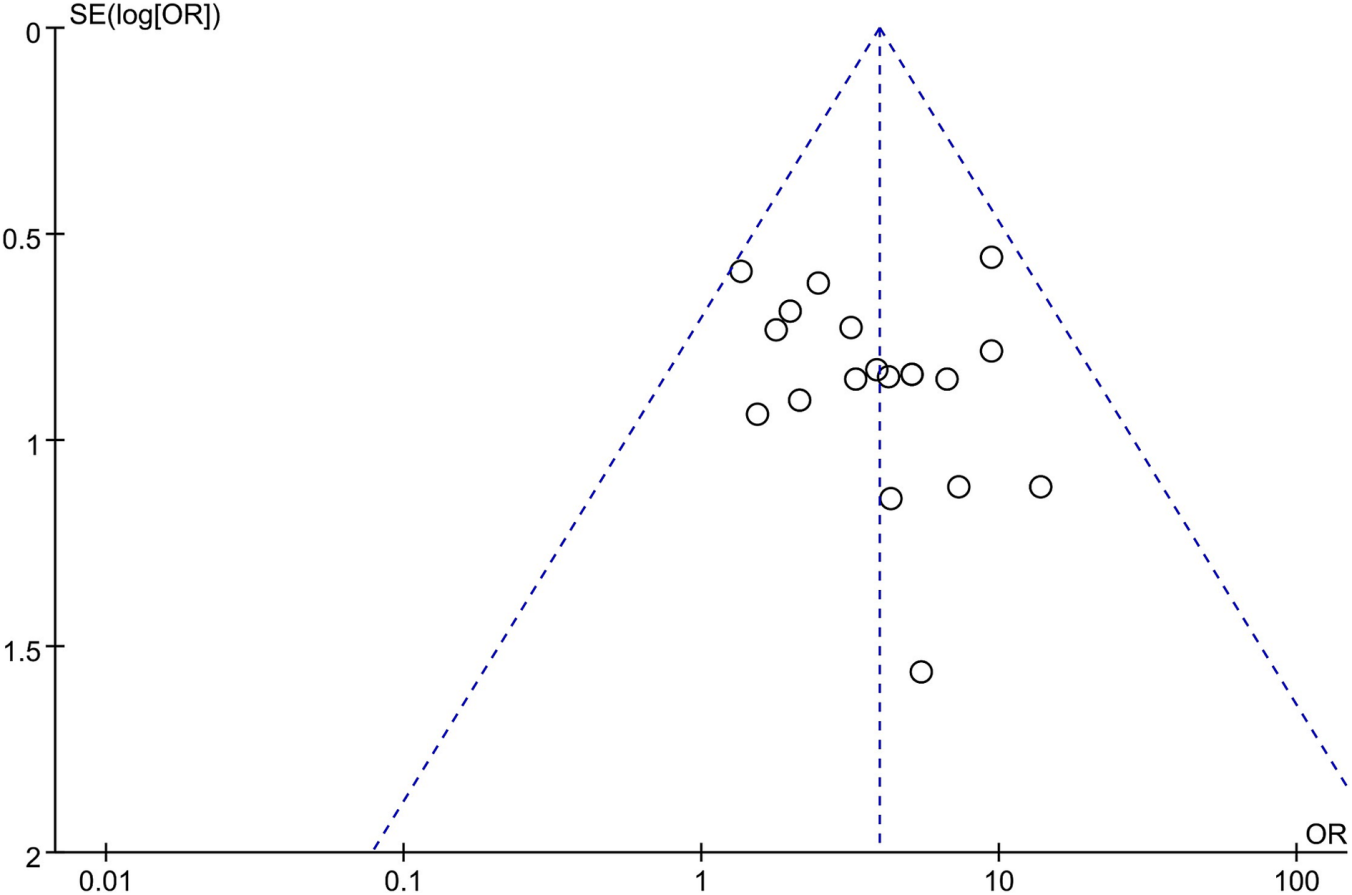
Funnel chart of total efficiency.

Publication bias was assessed by plotting a funnel plot of the total effectiveness rate of the outcome metrics for the most significant number of included studies. The 19 studies were distributed roughly symmetrically around the center line, suggesting that publication bias was insignificant and had a negligible effect on the amount of the combined impact (see Figure 10).

### 3.6 Reliability of the evidence

**Figure 11:**
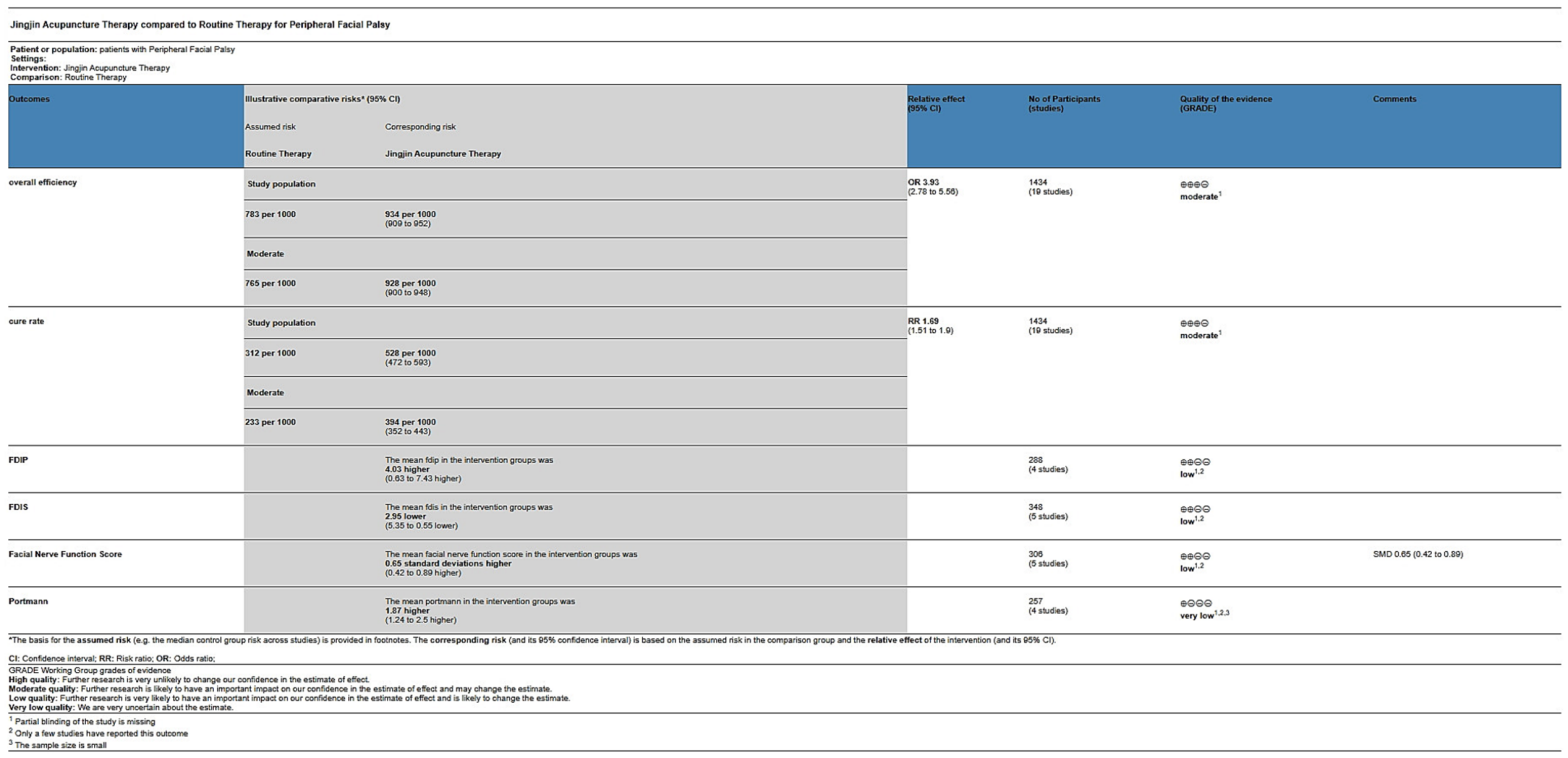
Level of evidence (GRADE).

The reliability of the evidence was assessed using the GRADEpro GDT, which showed a range of evidence levels from moderate to very low for all measures (see Figure 11).

## 4 Results and discussion

### 4.1 Summary

In this systematic evaluation of the clinical efficacy of the treatment of peripheral facial paralysis using Jingjin acupuncture therapy, a total of 1,436 cases in 19 studies were analyzed. The meta-analysis showed that the majority of the studies showed favorable trends.

All studies reported the overall effectiveness rate and cure rate, which are the most commonly tested indicators (38). The results showed that Jingjin acupuncture therapy significantly increased the overall effectiveness rate and cure rate of spasticity improvement. The improvement of spasticity helps to restore the normal function of facial muscles, reduce pain and discomfort, and improve patients’ quality of life. Jingjin acupuncture therapy may reduce inflammation and neuroedema by regulating the excitability of the nervous system and promoting local blood and lymphatic circulation.

The FDIP and FDIS scores are frequently used outcome measures, with four studies reporting FDIP scores and five studies reporting FDIS scores, and the results showed that Jingjin acupuncture therapy significantly increased the FDIP scores and decreased the FDIS scores. The high FDIP and low FDIS scores indicated significant improvement in the patient’s facial dysfunction and restoration of facial muscle mobility. In addition, the patients’ social function significantly improved; they were able to participate in social activities and improve their quality of life. Several studies have found that Jingjin acupuncture therapy could promote facial muscle recovery by upregulating neurotrophic factors, enhancing adhesion molecule expression, improving microcirculation, and promoting facial nerve recovery, which regulates nerve conduction and muscle excitability (39).

In assessing the effectiveness of Jingjin acupuncture therapy for cerebral palsy, not only were the overall efficacy rate, cure rate, FDIP, and FDIS scores considered but also several other measures, including the facial nerve function score and the Portmann scores, were also commonly used to assess peripheral facial palsy. It suggests that Jingjin acupuncture therapy can significantly improve the degree of peripheral facial paralysis of the patients, bodily function, social life function, and facial nerve function, and it provides evidence for treating peripheral facial paralysis patients. No studies reported adverse events, so we lack sufficient evidence to evaluate its safety.

### 4.2 Interpretation

For the disease of PFP, the treatment options of western medicine are limited, and there is often no way to deal with severe sequelae and refractory facial paralysis. Commonly used medications to treat PFP include prednisone, acyclovir, and methylcobalamin. Some scholars believe that PFP that is not healed for more than 3 months is called refractory facial paralysis (40). Therefore, early intervention and treatment are recommended to shorten the course of the disease (41). Clinically, PFP is treated with various therapeutic means with varying efficacy, and the majority of patients are more willing to receive TCM intervention than drug and surgical treatments (39).

With a long history of combining innovative and modern medicine, TCM therapies have evolved into essential complementary therapies that are considered safe, effective, and diverse (17). Acupuncture is a widely used complementary and alternative treatment that has been shown to have significant efficacy in peripheral neuropathies. The World Health Organization strongly recommends the use of acupuncture in the treatment of facial paralysis (42). Jingjin acupuncture therapy, as a type of acupuncture therapy, has been well documented for its usefulness in diagnosing and treating patients with peripheral facial palsy (43, 44). Jingjin acupuncture therapy promotes local blood circulation and tissue metabolism, lowers blood viscosity, regulates immune and inflammatory responses, loosens muscle spasms, regulates neurofactor secretion, and reduces inflammatory edema and pain (45). Jingjin acupuncture therapy can also widely stimulate the facial nerve, improve nerve nutrition, enhance the metabolism of nerve tissues, and increase their excitability. Thus, it can promote the recovery of facial nerve injuries (46).

### 4.3 Heterogeneity and potential heterogeneity analysis

The FDIP and FDIS scores show significant heterogeneity between the studies. In order to analyze the source of heterogeneity, sensitivity analysis was performed by excluding literature one by one, and if any literature was excluded, it still had high heterogeneity. The statistical results have not changed, indicating that single study is not the main source of heterogeneity in this study, indicating that the indicators of this study are stable, and the analysis may be due to the large differences in the course of disease, age and time in some studies. We then conducted subgroup analyses based on factors such as disease duration, sample size, and specific differences in interventions to assess the impact of these factors on the outcomes. However, there was no turnaround. This meta-analysis has differences in the control group’s setting. However, all of them used conventional therapies, and the specific implementation of the course of treatment, location, and means of rehabilitation was not the same. There was also a large difference in the number of cases in this study. Few studies were included in the FDIP, FDIS, facial nerve function, and Portmann score scales, which may increase the probability of bias.

### 4.4 Quality of evidence

The quality of evidence for the overall effectiveness rate and cure rate was moderate, and the quality of evidence for the FDIP, FDIS, Facial Nerve Function scores, and Portmann scores was low and very low, respectively. After discussion, our review panel concluded that the main reasons that led to the decrease in the level of evidence for this study were: 1. Partial blinding of the study was missing; 2. Only a few studies reported this outcome; and 3. The sample size was small. Therefore, although the results of this study suggest that Jingjin acupuncture therapy is beneficial for patients with peripheral facial paralysis, we still need randomized controlled trials using high-quality methodology to further improve the level of evidence.

### 4.5 Strengths and limitations

This is the first meta-analysis of the efficacy of Jingjin acupuncture therapy for peripheral facial palsy, and all RCTs were comprehensively screened simultaneously to ensure accuracy. A comprehensive set of outcome metrics was included to comprehensively evaluate the efficacy of Jingjin acupuncture therapy for peripheral facial palsy. Finally, the GRADE evaluation method was applied to analyze the therapeutic efficacy of Jingjin acupuncture therapy more accurately and objectively. However, the following limitations exist in this study:

First, the 19 studies included in the meta-analysis were all Chinese studies, with no foreign studies, so the differences in implementation between domestic and foreign countries could not be evaluated more comprehensively. Second, the overall sample sizes of some of the studies were small. There were no large-sample or multicenter studies. The random allocation methods in some studies were not described in detail. Third, only four studies involved allocation concealment or blinded designs, which may have exaggerated clinical treatment effects. Fourth, the majority of the studies did not conduct follow-ups to observe long-term efficacy. Fifth, some studies had unequal sample sizes in the control and experimental groups, which may pose a risk of bias. Sixth, some studies were of low quality, and the interventions or testing indicators during the study may have been inaccurate. Seventh, some studies had greater heterogeneity, which may affect the reliability of the conclusions and need to be treated with caution. Eighth, some studies did not report adverse conditions. The above shortcomings may have a certain impact on the evaluation results and limit the strength of the argument of this study to a certain extent.

### 4.6 Enlightenment

1. Standardize the clinical diagnostic criteria, inclusion criteria, intervention criteria, and efficacy criteria; 2. Design reasonable and scientific, carry out high-quality multi-center, large-sample randomized controlled clinical trials, and pay attention to describing the method of random allocation, allocation concealment, blinding, adverse reactions, and follow-up; 3. Study in depth the timing, selection, depth, intensity, manipulation and duration of acupuncture points, to make the treatment more scientific and standardized; 4. The principles and methods of clinical epidemiology and evidence-based medicine should be strictly followed, so that the research results can better guide the clinical practice.

## 5 Conclusion

Jingjin acupuncture therapy has demonstrated significant efficacy in the treatment of peripheral facial paralysis and is of great value in clinical promotion. Compared to existing treatments, Jingjin acupuncture therapy has demonstrated superior results in improving patients’ facial function and quality of life. However, the design and operation of the current study need to be more scientific and standardized. To provide more reliable clinical evidence, there is an urgent need for multicenter, large-sample, high-quality randomized controlled studies. These studies should focus on the following specific areas and directions: 1. The effect of disease durations on efficacy: to explore the response of patients with different disease durations to Jingjin acupuncture therapy to determine the optimal timing of treatment; 2. Specific differences in interventions: to study the effect of different combinations of acupoints and depth of acupuncture on the treatment efficacy and to optimize the treatment regimen; 3. The effect of patient characteristics is to analyze the different ages, genders, and severity of disease of the patient’s response to treatment and develop personalized treatment strategies; and 4. Long-term efficacy and safety: to assess the long-term efficacy and safety of Jingjin acupuncture therapy and ensure its sustainability in clinical application. Through these high-quality studies, we can further validate the clinical application value of Jingjin acupuncture therapy and provide a solid scientific basis for its promotion in treating peripheral facial palsy.

## Data Availability

The datasets presented in this study can be found in online repositories. The names of the repository and accession number(s) can be found at: China knowledge network database (https://www.cnki.net/).
